# Rosiglitazone-Induced Mitochondrial Biogenesis in White Adipose Tissue Is Independent of Peroxisome Proliferator-Activated Receptor γ Coactivator-1α

**DOI:** 10.1371/journal.pone.0026989

**Published:** 2011-11-07

**Authors:** Rosario Pardo, Natàlia Enguix, Jaime Lasheras, Juan E. Feliu, Anastasia Kralli, Josep A. Villena

**Affiliations:** 1 Laboratory of Metabolism and Obesity, Unit of Diabetes and Metabolism, Vall d'Hebron-Institut de Recerca, Universitat Autònoma de Barcelona, Barcelona, Spain; 2 Department of Chemical Physiology, The Scripps Research Institute, La Jolla, California, United States of America; Institut de Génomique Fonctionnelle de Lyon, France

## Abstract

**Background:**

Thiazolidinediones, a family of insulin-sensitizing drugs commonly used to treat type 2 diabetes, are thought to exert their effects in part by promoting mitochondrial biogenesis in white adipose tissue through the transcriptional coactivator PGC-1α (Peroxisome Proliferator-Activated Receptor γ Coactivator-1α).

**Methodology/Principal Findings:**

To assess the role of PGC-1α in the control of rosiglitazone-induced mitochondrial biogenesis, we have generated a mouse model that lacks expression of PGC-1α specifically in adipose tissues (PGC-1α-FAT-KO mice). We found that expression of genes encoding for mitochondrial proteins involved in oxidative phosphorylation, tricarboxylic acid cycle or fatty acid oxidation, was similar in white adipose tissue of wild type and PGC-1α-FAT-KO mice. Furthermore, the absence of PGC-1α did not prevent the positive effect of rosiglitazone on mitochondrial gene expression or biogenesis, but it precluded the induction by rosiglitazone of UCP1 and other brown fat-specific genes in white adipose tissue. Consistent with the *in vivo* findings, basal and rosiglitazone-induced mitochondrial gene expression in 3T3-L1 adipocytes was unaffected by the knockdown of PGC-1α but it was impaired when PGC-1β expression was knockdown by the use of specific siRNA.

**Conclusions/Significance:**

These results indicate that in white adipose tissue PGC-1α is dispensable for basal and rosiglitazone-induced mitochondrial biogenesis but required for the rosiglitazone-induced expression of UCP1 and other brown adipocyte-specific markers. Our study suggests that PGC-1α is important for the appearance of brown adipocytes in white adipose tissue. Our findings also provide evidence that PGC-1β and not PGC-1α regulates basal and rosiglitazone-induced mitochondrial gene expression in white adipocytes.

## Introduction

Type 2 diabetes is strongly associated with a decrease in mitochondrial mass and function [1]. Clinical studies show that individuals with insulin resistance or type 2 diabetes have reduced expression of mitochondrial genes in skeletal muscle and adipose tissue [2], [3], [4], [5], [6], [7]. Consistent with decreased gene expression, insulin-resistant offspring of patients with type 2 diabetes exhibit diminished mitochondrial oxidative capacity [8]. Similarly, impaired mitochondrial biogenesis and function in adipose tissue is also seen in animal models of type 2 diabetes [9], [10], [11]. While it is not clear to what extent impaired mitochondrial function is an underlying cause or a consequence of insulin resistance, it is striking that both life style interventions (i.e. exercise or calorie restriction) and pharmacological treatments (i.e. thiazolidinediones) that enhance oxidative metabolism are also effective in ameliorating whole body insulin sensitivity, an effect that has been associated with the capacity of such treatments to induce mitochondrial biogenesis [12], [13], [14], [15].

The insulin-sensitizing properties of thiazolidinediones (TZDs), a family of PPARγ agonist compounds used to treat type 2 diabetes, have been traditionally attributed to their ability to enhance adipogenesis, via activation of PPARγ [16]. However, compelling evidence suggests that TZDs may also contribute to insulin sensitivity by affecting energy expenditure pathways. Supporting this hypothesis, pioglitazone and rosiglitazone, two commonly used TZDs, have been shown to induce mitochondrial biogenesis and fatty acid oxidation in human adipose tissue [2], [17]. Likewise, rosiglitazone treatment leads to increased mitochondrial gene expression in the adipose tissue of ob/ob and db/db diabetic mice [10], [18]. However, the mechanisms by which TZDs promote mitochondrial gene expression and biogenesis in adipose tissue are not well understood.

Several transcription factors, including Nuclear Respiratory Factor 1 (NRF-1), GA-binding protein (Gabp/NRF-2), the Peroxisome Proliferator-Activated Receptors PPARα and PPARδ, and the Estrogen-Related Receptors ERRα and ERRγ have been identified as regulators of the expression of oxidative phosphorylation (OxPhos), fatty acid oxidation (FAO) and mitochondrial biogenesis genes (reviewed in [19], [20]). The activity of these transcription factors is coordinated by the coactivators PGC-1α and β, which play a pivotal role in the regulation of energy metabolism by integrating diverse environmental and physiological cues that signal energy needs and promoting mitochondrial biogenesis [21]. Interestingly, the expression of PGC-1α is reduced in the skeletal muscle and adipose tissue of diabetic patients [2], [5], [7], suggesting that decreased PGC-1α underlies a consequent impairment in mitochondrial function in type 2 diabetes. Conversely, a positive correlation between mitochondrial biogenesis induced by TZDs and PGC-1α expression has been documented in white adipose tissue (WAT) [2], [10], [18]. This has led to the notion that TZDs may exert their beneficial effects on insulin sensitivity in part by promoting mitochondrial biogenesis and energy expenditure in WAT through PGC-1α. However, the relevance of PGC-1α in supporting basal or TZD-induced mitochondrial biogenesis in WAT has not yet been addressed.

To study the role of PGC-1α on mitochondrial biogenesis in WAT, we have generated a mouse model that lacks expression of PGC-1α coactivator specifically in adipocytes. Our results show that PGC-1α is dispensable for basal and rosiglitazone-induced mitochondrial biogenesis and function. Consistent with the known role of PGC-1α in brown adipose tissue (BAT), PGC-1α in WAT regulates a small subset of genes involved in adaptive thermogenesis, suggesting that PGC-1α is important for the appearance of brown adipocytes in WAT in response to rosiglitazone. We also provide *in vitro* evidence for a role of PGC-1β in the control of basal and rosiglitazone-induced expression of mitochondrial genes in white adipocytes.

## Results

### Characterization of PGC-1α-FAT-KO mice

**Figure 1:**
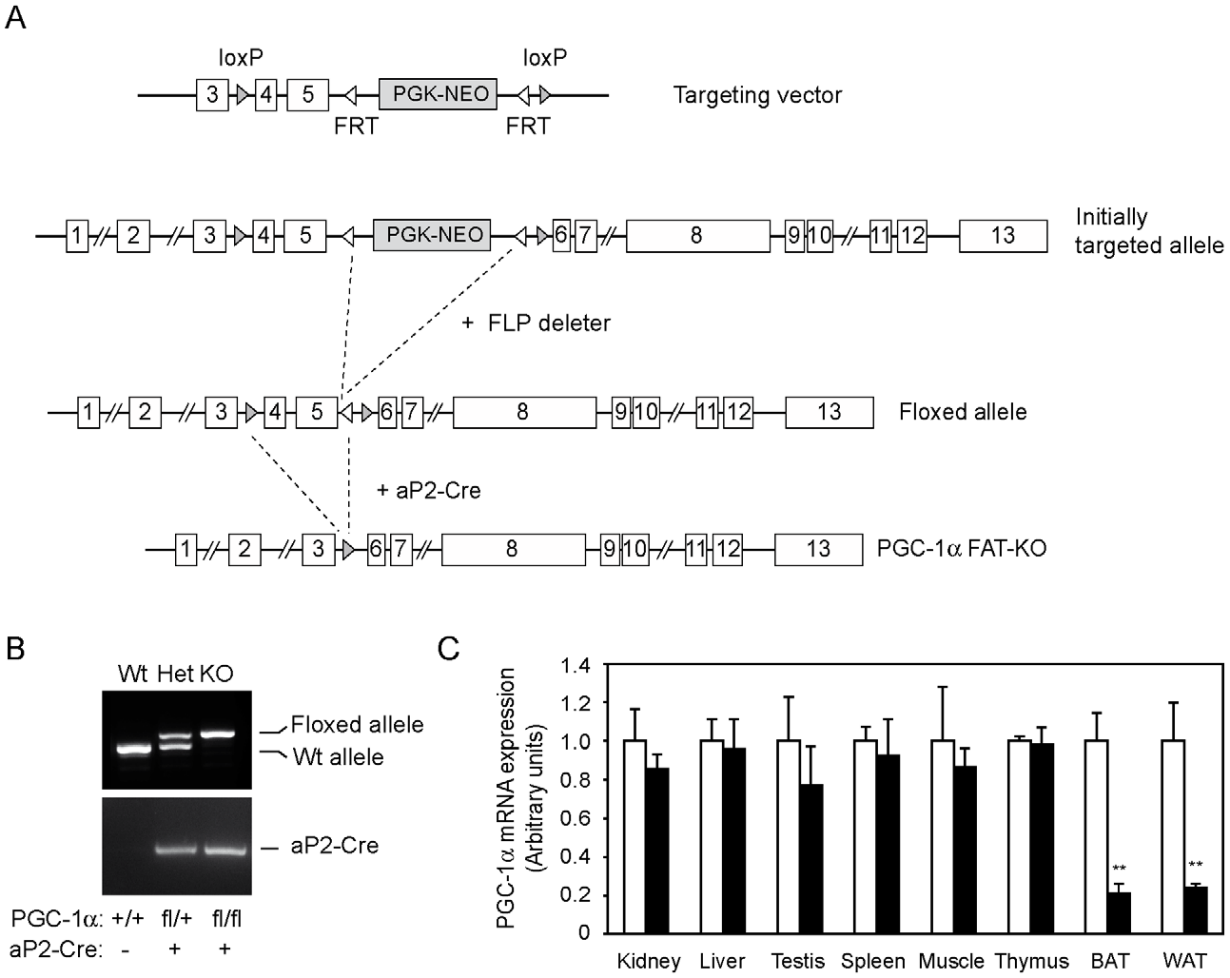
Generation of adipose-specific PGC-1α knockout mice. (A) A targeting vector, containing a PGK-NEO selection cassette flanked by FRT sites in intron 5 and having exons 4 and 5 of *Ppargc1*a gene flanked by loxP sites, was used to generate mice with floxed *Ppargc1a* alleles. PGC-1α-FAT-KO mice were generated by crossing mice with floxed *Ppargc1a* alleles to aP2-Cre mice that overexpress Cre recombinase in adipose tissues under the control of the adipocyte-specific aP2 promoter. (B) PCR of tail genomic DNA was used to detect loxP flanked *Ppargc1a* alleles and the presence of aP2-Cre transgene. Mice homozygous for the loxP-flanked allele and positive for the aP2-Cre transgene are referred to as fat-specific PGC-1α knockout mice (PGC-1α-FAT-KO). (C) Expression of PGC-1α mRNA was analyzed by real-time quantitative PCR in several tissues of Wt (white bars) or PGC-1α-FAT-KO (black bars) mice. Values are mean ± SEM. **P<0.01, n = 4–5 animals/group.

To investigate the role of PGC-1α in white adipocytes, we first generated a mouse model in which the *Ppargc1a* gene was disrupted by homologous recombination specifically in adipose tissue (PGC-1α-FAT-KO mice). For this purpose, we crossed mice that harbor loxP sites flanking exons 4 and 5 of the *Ppargc1a* gene with aP2-Cre transgenic mice (Figure 1A and 1B). As shown in Figure 1C, adipocyte-specific Cre recombinase-driven disruption of the *Ppargc1a* gene resulted in a dramatic decrease in the expression of PGC-1α mRNA in adipose tissues, both white and brown, but not in other tissues. We then raised PGC-1α-FAT-KO and littermate mice at thermoneutrality (30°C), to minimize the activation of BAT and the effects of decreased PGC-1α activity and defective adaptive thermogenesis in brown adipocytes [22]. At this temperature, brown adipocytes adopt a white adipocyte-like appearance, with big unilocular lipid droplets (Figure S1).

**Figure 2:**
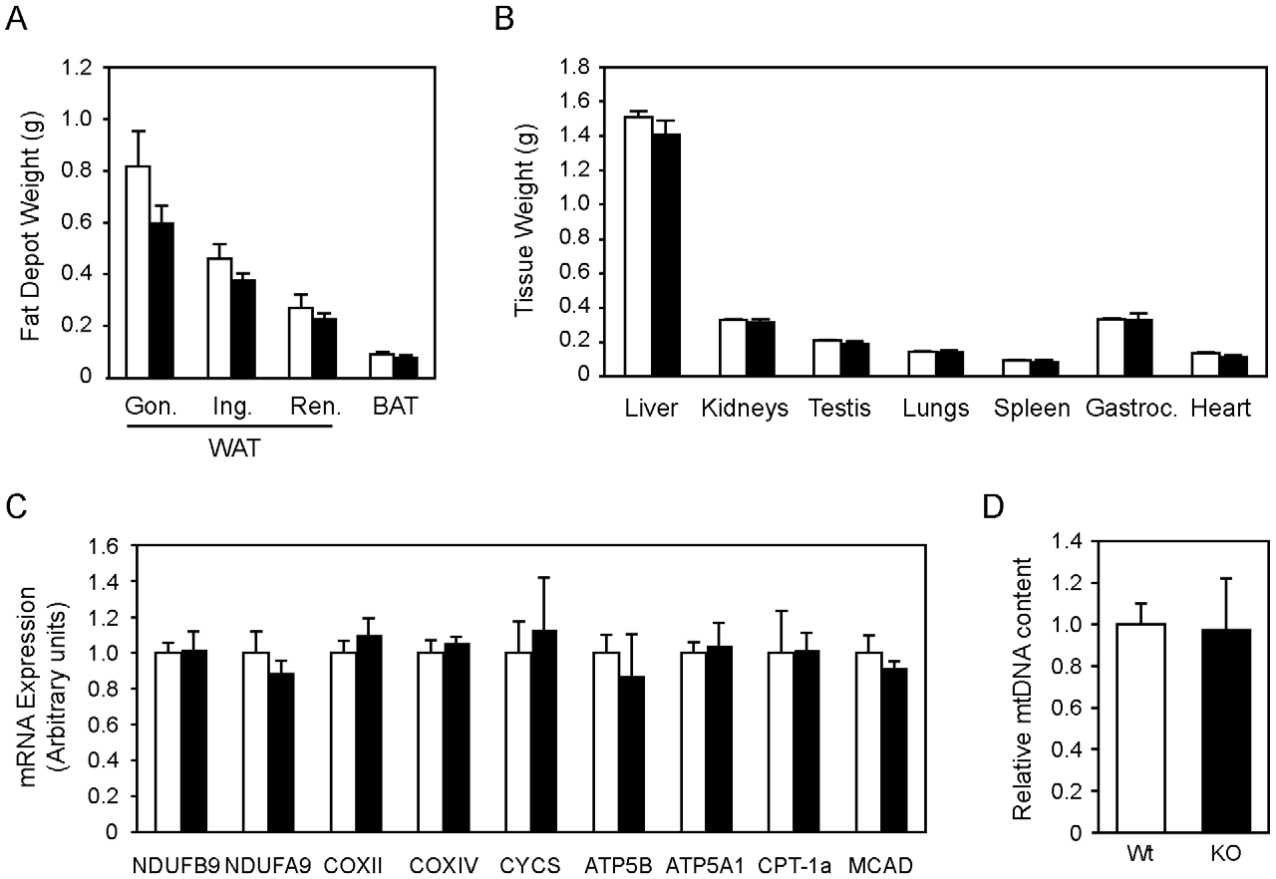
Characterization of PGC-1α-FAT-KO mice. (A) Mass of major white and brown adipose tissues of 16-week old Wt (white bars) and PGC-1α-FAT-KO (black bars) mice fed a regular diet (Gon., visceral gonadal; Ing., subcutaneous inguinal; Ren., visceral retroperitoneal; BAT, interescapular brown adipose tissue) (n = 9–10 animals/group). (B) Weight of tissues of Wt and PGC-1α-FAT-KO mice (Gastroc., gastrocnemius muscle). (C) mRNA expression of mitochondrial genes of the OxPhos system or genes involved in fatty acid oxidation was determined by real-time quantitative PCR. (D) Mitochondrial mass in WAT was estimated by measuring mtDNA content by quantitative real-time PCR. Results are expressed as mean ± SEM. (n = 5 animals/group).

Under these housing conditions, PGC-1α-FAT-KO male mice fed a standard chow diet weighed slightly less than their Wt littermates at weaning (Wt = 13.04±0.7, n = 11, vs. PGC-1α-FAT-KO = 10.9±0.6, n = 9; *P* = 0.08). A tendency towards lower body weight in PGC-1α-FAT-KO mice was maintained through the duration of the experimental period (Wt = 33.0±0.8, n = 11, vs. PGC-1α-FAT-KO = 31.7±0.6, n = 9; *P* = 0.39 at 16 weeks), but differences did not reach statistical significance. Consistent with a lower body weight, adult PGC-1α-FAT-KO mice exhibited a modest, not significant reduction in the weight of major adipose tissue depots (Figure 2A), while the weight of other tissues were comparable between Wt and PGC-1α-FAT-KO (Figure 2B).

PGC-1α regulates mitochondrial biogenesis in a wide variety of tissues by coordinately regulating mitochondrial gene expression [23]. However, expression of characterized PGC-1α-responsive genes in PGC-1α-FAT-KO, such as genes of the OxPhos system (NDUFB9, NDUFA9, COXII, COXIV, CYCS, ATP5B, ATP5A1) or genes involved in fatty acid oxidation (CPT-1a, MCAD), were not affected by the lack of PGC-1α (Figure 2C). Also, mtDNA content was similar in Wt and PGC-1α-FAT-KO mice (Figure 2D). These results suggest that PGC-1α is not required for basal mitochondrial gene expression or biogenesis in WAT.

### Mitochondrial biogenesis induced by TZDs is independent of PGC-1α

Rosiglitazone, like other PPARγ agonists, induces mitochondrial biogenesis in WAT by mechanisms that are not fully understood [2], [10], [17], [18]. It has been suggested that the effects of rosiglitazone and other TZDs on mitochondrial biogenesis and insulin sensitivity could be mediated in part by PGC-1α, since amelioration of insulin resistance and induction of mitochondrial gene expression by TZDs is paralleled by an increase in PGC-1α levels [2], [10], [17]. To test this hypothesis, we first fed PGC-1α-FAT-KO mice and Wt littermates a high fat diet (HFD) to induce insulin resistance and then treated them with rosiglitazone for 15 days.

**Figure 3:**
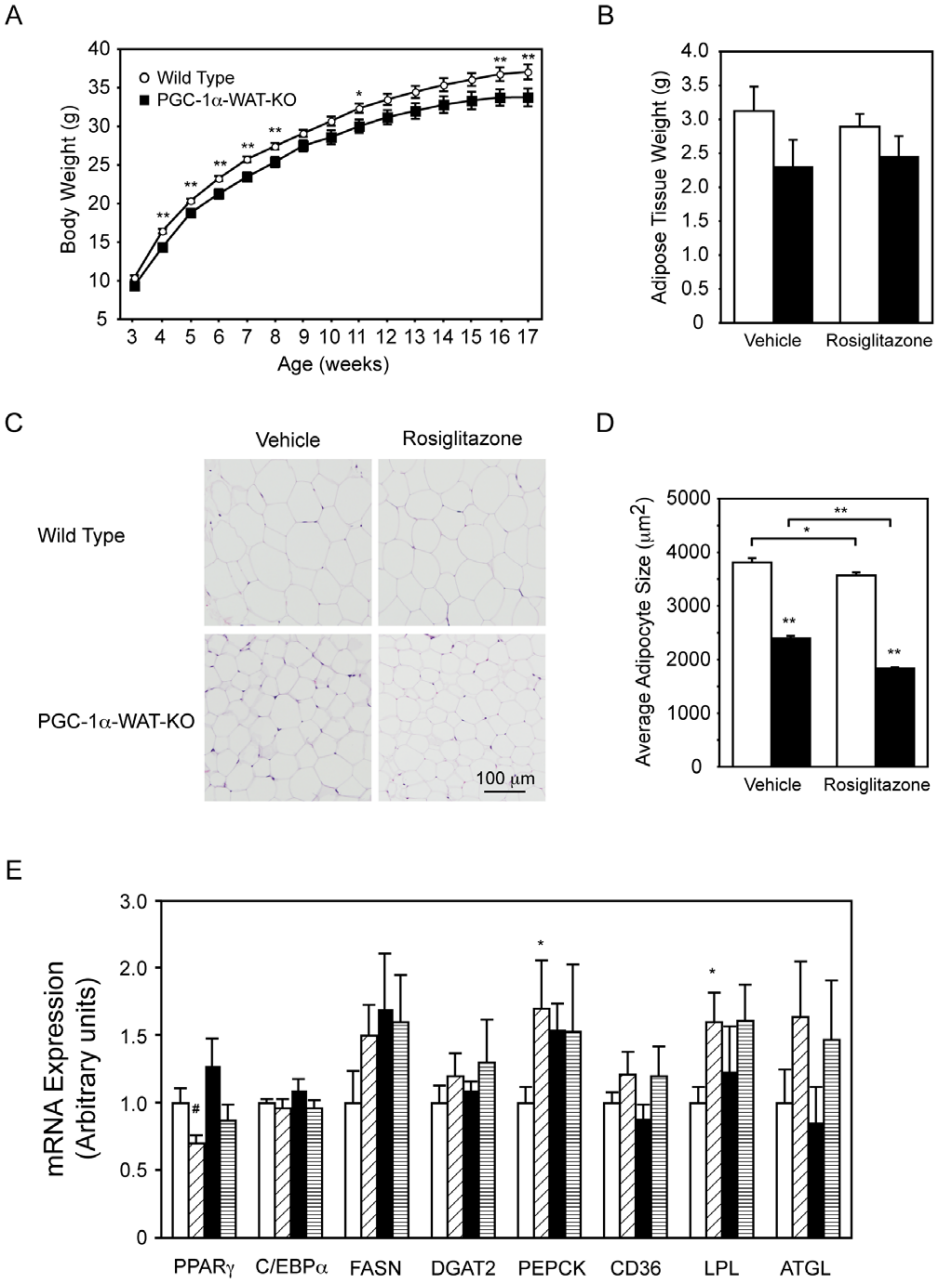
Reduced body weight and adipose tissue mass in PGC-1α-FAT-KO mice fed a high fat diet. (A) Body weight of PGC-1α-FAT-KO mice and Wt littermates housed at thermoneutrality and fed a high fat diet (n = 14–18 animals/group). (B) Weight of all major white adipose depots in mice treated with vehicle or rosiglitazone (n = 5–7 animals/group). (C) Histological analysis of subcutaneous WAT of Wt and PGC-1α-FAT-KO mice treated with vehicle or rosiglitazone. (D) Measurement of adipocyte size was performed using Image J software and at least 300 cells from 3 different sections were measured (n = 3 animals/group). (E) mRNA expression of genes involved in adipocyte differentiation or triglyceride metabolism was measured in WAT by real time quantitative PCR. Results are expressed as mean ± SEM. (n = 5 animals/group) ^#^, *P<0.05, **P<0.01. 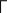, Wt+vehicle; 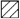, Wt+rosiglitazone; 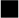, PGC-1α-FAT-KO+vehicle; 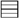, PGC-1α-FAT-KO+rosiglitazone.

PGC-1α-FAT-KO mice fed a HFD weighed less than Wt littermates (Figure 3A), a difference in body weight that correlated with decreased adipose tissue mass (Figure 3B). A slight reduction in liver weight, but not in skeletal muscles, was also observed (Wt = 1.25±0.06 vs PGC-1α-FAT-KO = 1.09±0.06, *P* = 0.051), which could also contribute to reduced body mass. Histological analysis of WAT revealed that adipocytes were significantly smaller in PGC-1α-FAT-KO than in Wt, which may account for the reduction in WAT mass (Figure 3C-D). Likewise, brown adipocytes from interescapular BAT of PGC-1α-FAT-KO mice were smaller and practically indistinguishable from white adipocytes due to the large accumulation of triglycerides (Figure S2). The decrease in adipocyte size could not be attributed to an impairment of adipocyte differentiation, since expression of mature adipocyte markers, such as PPARγ or C/EBPα was similar in PGC-1α-FAT-KO and Wt mice (Figure 3E). Similarly, we did not observed significant differences between PGC-1α-FAT-KO and Wt mice in the expression of genes involved in diverse aspects of triglyceride metabolism, such as synthesis (FASN, DGAT, PEPCK), transport (CD36) or hydrolysis (LPL, ATGL) (Figure 3E).

Treatment with rosiglitazone notably improved the metabolic profile of high fat diet fed Wt mice, significantly decreasing triglycerides, free fatty acids, cholesterol, glucose and insulin levels (Table 1). Consistent with the amelioration of the serological metabolic profile, rosiglitazone also improved glucose tolerance and insulin sensitivity (Figure S3). Notably, we observed no significant differences in serum parameters, glucose tolerance or insulin sensitivity between Wt and PGC-1α-FAT-KO mice, in the absence or presence of rosiglitazone treatment. These results suggest that PGC-1α in WAT does not affect whole body glucose or lipid homeostasis and is not required for an effective metabolic response to rosiglitazone.

**Table 1:** Serum parameters in 5 h-fasted Wt and PGC-1α-FAT-KO mice treated with vehicle or rosiglitazone.

	Vehicle		Rosiglitazone	
	Wild Type	PGC-1α-WAT-KO	Wild Type	PGC-1α-WAT-KO
Glucose (mg/dL)	130.7±5.1	122.0±7.1	99.4±3.9**	99.2±3.6*
FFA (mmol/L)	0.640±0.09	0.558±0.06	0.416±0.01*	0.478±0.05
Triglycerides (mg/dL)	71.2±5.6	63.5±4.3	47.1±2.8**	47.8±3.2*
Cholesterol (mg/dL)	250.0±9.3	205.9±27.4	199.6±14.7*	185.9±21.3
Insulin (ng/ml)	1.95±0.29	1.55±0.31	0.95±0.17**	0.91±0.30

*P<0.05;

**P<0.01, n = 7–10 animals/group.

**Figure 4:**
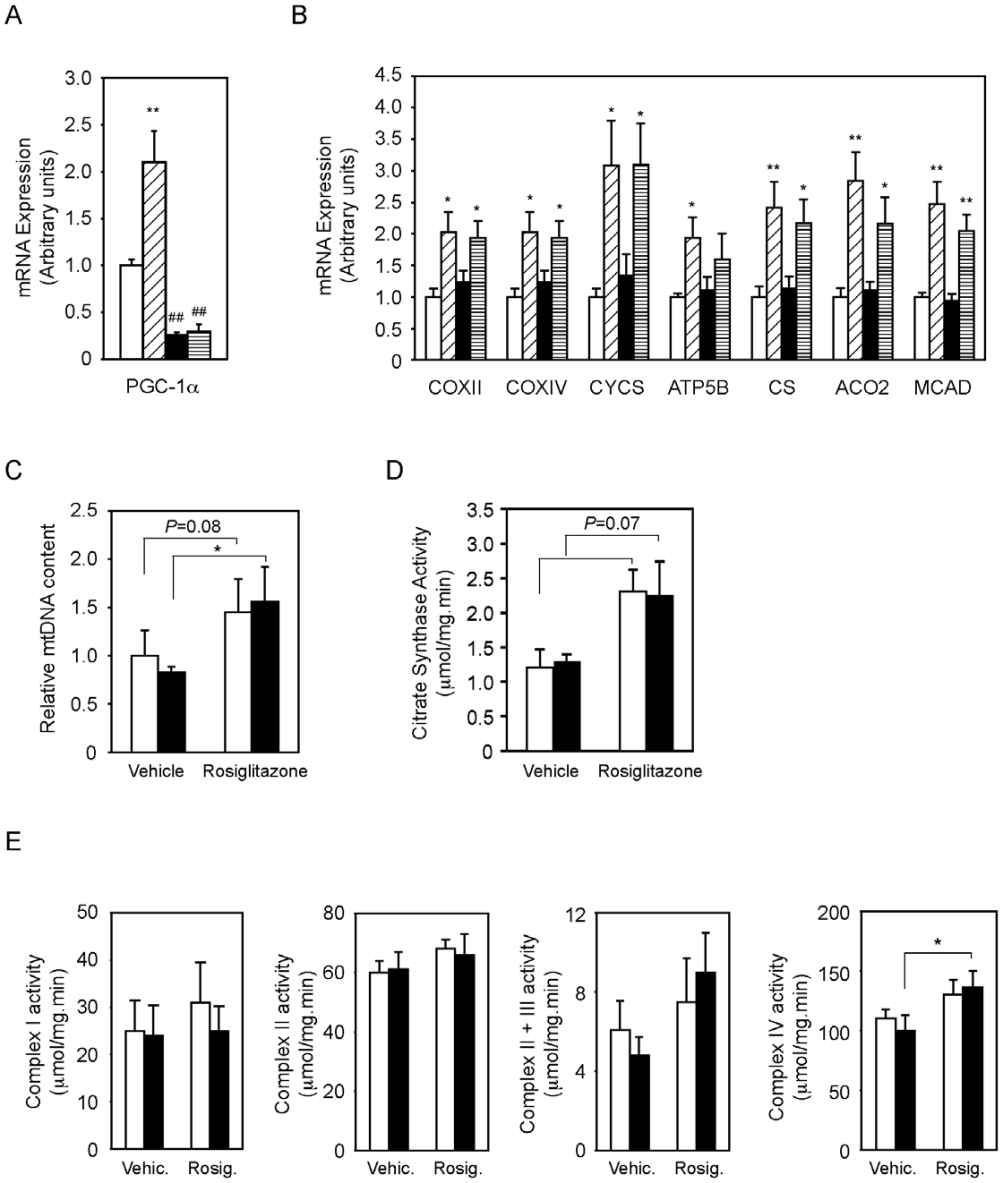
Rosiglitazone-induced mitochondrial gene expression and function in PGC-1α-FAT-KO mice. Expression of PGC-1α (A) or mitochondrial genes (B) was analyzed by real-time quantitative PCR in retroperitoneal WAT of Wt or PGC-1α-FAT-KO mice subjected to vehicle or rosiglitazone treatment. (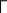, Wt+vehicle; 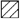, Wt+rosiglitazone; 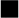, PGC-1α-FAT-KO+vehicle; 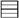, PGC-1α-FAT-KO+rosiglitazone.) (C) Mitochondrial mass in WAT was estimated by measuring mtDNA content by real-time PCR. (D) Citrate synthase activity was measured in WAT crude extracts to assess overall mitochondrial function. (E) Activity of mitochondrial respiratory chain complexes was measured in mitochondria-enriched fractions from WAT by spectrophotometric methods. Results are expressed as mean ± SEM. (n = 5-8 animals/group). *P<0.05, **P<0.01. In panels C, D and E, Wt mice are indicated as white bars and PGC-1α-FAT-KO as black bars.

As previously reported, treatment of Wt mice with rosiglitazone increased PGC-1α expression in WAT ∼2 fold (Figure 4A). The effect of rosiglitazone on PGC-1α expression was completely blunted in PGC-1α-FAT-KO mice (Figure 4A). In Wt mice, the increase in PGC-1α mRNA levels was paralleled by a rise in the expression of genes encoding for mitochondrial proteins of the OxPhos system (COXII, COXIV, CYCS, ATP5B), tricarboxylic acid cycle (CS and ACO2) and FAO (MCAD) (Figure 4B). Rosiglitazone also led to an increase in mitochondrial DNA content and citrate synthase activity in Wt mice (Figure 4C–D). Surprisingly, the lack of PGC-1α did not prevent the rosiglitazone-induced expression of OxPhos, TCA and FAO genes, or the increase in mitochondrial biogenesis and citrate synthase activity (Figure 4B–D). Consistent with the similar expression levels of mitochondrial genes, Wt and PGC-1α-FAT-KO mice showed comparable activities of mitochondrial respiratory chain complexes (Figure 4E). In summary, rosiglitazone was as effective in Wt as in PGC-1α-FAT-KO mice, suggesting that PGC-1α is not required for the rosiglitazone-induced mitochondrial biogenesis or function in WAT.

### PGC-1α regulates rosiglitazone-induced expression of brown adipocyte-specific mitochondrial genes

PPARγ agonists have also been shown to induce the expression of brown adipocyte-specific markers, such as UCP1, in WAT. Since PGC-1α regulates the expression of these same genes in BAT, we next asked if PGC-1α could play a role in this aspect of the rosiglitazone response.

**Figure 5:**
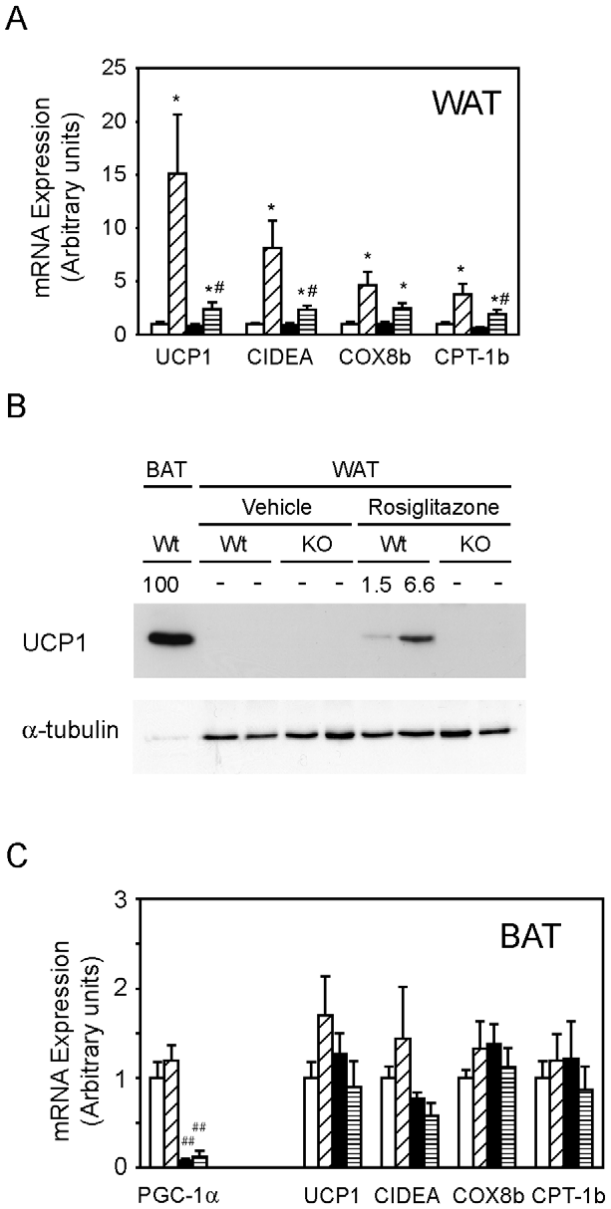
Effect of the lack of PGC-1α on brown adipocyte-specific genes in WAT of PGC-1α-FAT-KO mice. (A) Expression of brown adipocyte-specific genes was determined by real-time quantitative PCR in retroperitoneal WAT of Wt and PGC-1α-FAT-KO mice. (B) UCP1 protein levels were detected by western blot in WAT of Wt and PGC-1α-FAT-KO mice treated with vehicle or rosiglitazone. BAT protein extract from Wt mice housed at 21°C was used as a positive control. Numbers indicate UCP1/α-tubulin signal ratio expressed as values relative to the positive control, which was given an arbitrary value of 100. (C) Expression of brown-specific genes in interescapular BAT was analyzed by real time quantitative PCR. Results are expressed as mean ± SEM. (n = 5–8 animals/group). ^#^ indicates statistical significance of the comparison between Wt and PGC-1α-FAT-KO mice; * indicates statistical significance of the comparison between vehicle- and rosiglitazone-treated groups. ^#^,*P<0.05, **P<0.01. 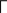 , Wt+vehicle; 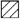 , Wt+rosiglitazone; 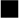 , PGC-1α-FAT-KO+vehicle; 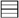 , PGC-1α-FAT-KO+rosiglitazone.

Rosiglitazone treatment of Wt mice increased mRNA levels of UCP1 and other brown adipocyte markers such as CIDEA, COX8b or CPT1b by 4- to 15-fold in WAT (Figure 5A). The induction of these genes was blunted in PGC-1α-FAT-KO, indicating that PGC-1α is required to achieve maximal induction of brown fat-specific genes by TZDs in WAT. Consistent with the mRNA expression data, induction of UCP1 protein levels by rosiglitazone was blunted in WAT of PGC-1α-FAT-KO (Figure 5B).

Interestingly, we found that lack of PGC-1α in BAT did not affect basal expression of UCP1 or other brown adipocyte-specific genes when mice were maintained at thermoneutrality (Figure 5C). Moreover, we also found that rosiglitazone had no effect (CPT-1b and COX8b) or minimal effects (UCP1 and CIDEA) on the expression of these genes in interescapular BAT (Figure 5C). These findings suggest that cells expressing UCP1 and other brown fat markers in WAT may respond differently to rosiglitazone than brown adipocytes of the interescapular brown fat tissue.

### PGC-1β regulates basal and rosiglitazone-induced expression of mitochondrial genes in cultured white adipocytes

**Figure 6:**
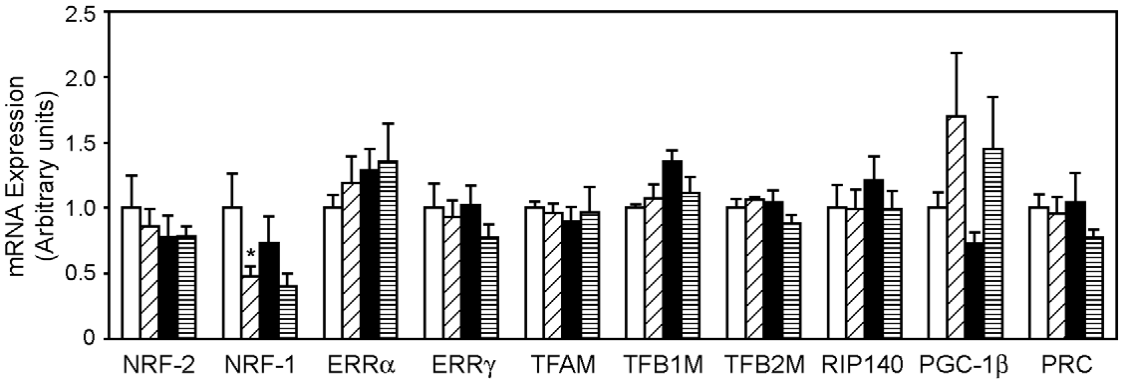
Expression of mitochondrial biogenesis regulators in WAT of PGC-1α-FAT-KO mice. Real-time quantitative PCR was used to determine expression levels of major regulators of mitochondrial gene expression in WAT of Wt and PGC-1α-FAT-KO mice. Results are expressed as mean ± SEM. (n = 5–8 animals/group). *P<0.05. 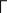, Wt+vehicle; 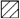, Wt+rosiglitazone; 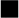, PGC-1α-FAT-KO+vehicle; 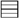, PGC-1α-FAT-KO+rosiglitazone.

The lack of a PGC-1α role in rosiglitazone-induced mitochondrial biogenesis raises the question of how rosiglitazone exerts its effects on this process. To address this question, we first checked expression of other transcriptional regulators of mitochondrial biogenesis in Wt and PGC-1α-FAT-KO, under basal and rosiglitazone-stimulated conditions. Among the transcriptional regulators analyzed, expression of the coactivator PGC-1β was induced 2-fold by rosiglitazone in WAT of both Wt and PGC-1α-FAT-KO mice (Figure 6), although changes did not reach statistical significance. The expression of other transcription factors known to participate in the regulation of mitochondrial gene expression (NRF-2, ERRs, TFAM, etc.) remained unchanged, with the exception of NRF-1 that was decreased by rosiglitazone. The fact that PGC-1β expression correlated with that of mitochondrial genes prompted us to hypothesize that PGC-1β could mediate the response to rosiglitazone.

**Figure 7:**
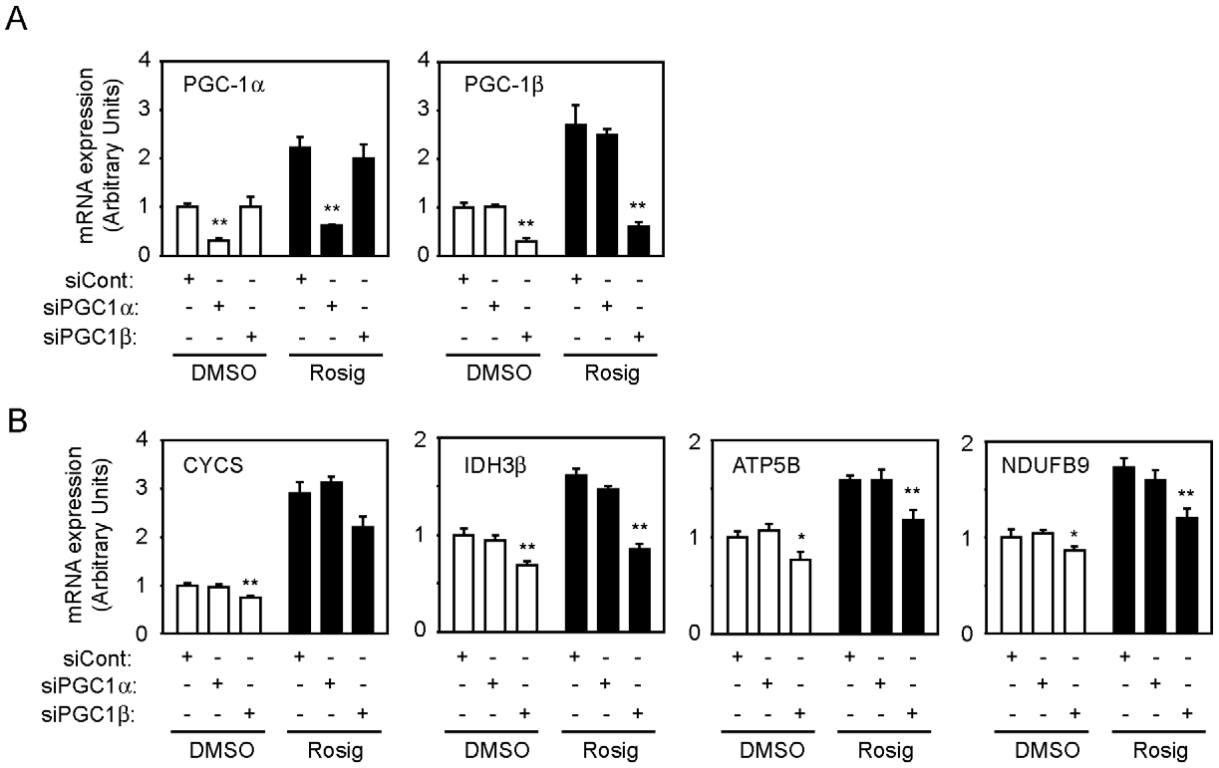
PGC-1β regulates mitochondrial gene expression in 3T3-L1 adipocytes. (A) PGC-1α and PGC-1β expression in 3T3-L1 adipocytes transfected with siRNAs specifically targeting PGC-1α or PGC-1α. (B) Expression of mitochondrial genes CYCS, IDH3β, ATP5B and NDUFB9 in adipocytes transfected with siRNA for PGC-1α or PGC-1β knockdown or siRNA control, and treated with vehicle (DMSO) or rosiglitazone (Rosig). Results are expressed as mean ± SEM of 3 independent experiments with triplicates. *P<0.05, **P<0.01.

**Figure 8:**
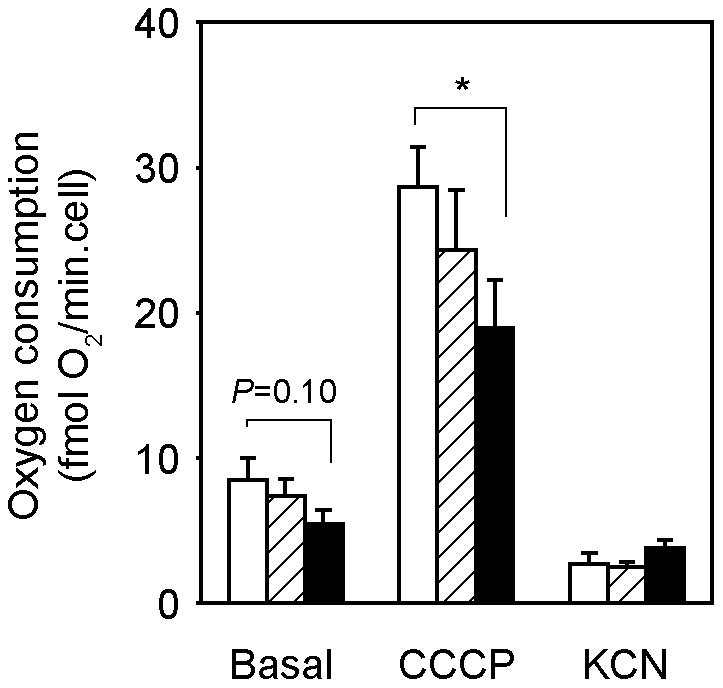
Down-regulation of PGC-1β decreases adipocyte respiration. 3T3-L1 adipocytes were transfected with siRNAs specifically targeting PGC-1α or PGC-1β or a control siRNA. Forty-eight hours after transfection, basal and maximal respiration (CCCP) was measured using a Clark-type oxygen electrode. Background oxygen consumption was determined after inhibiting mitochondrial respiration with 1 mM of KCN. Results are expressed as mean ± SEM of 3 independent experiments with triplicates. *P<0.05. 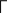, Control siRNA; 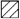, PGC-1α siRNA; 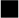, PGC-1β siRNA.

To investigate the role of PGC-1β on the rosiglitazone-induced expression of mitochondrial genes, we transfected 3T3-L1 adipocytes with siRNAs targeting PGC-1α or PGC-1β. The PGC-1α siRNA specifically knocked down PGC-1α expression by 70–80%, compared to adipocytes transfected with a non-target siRNA (Figure 7A). Consistent with our previous findings in PGC-1α-FAT-KO mice, reduced PGC-1α expression did not have any effect on the expression of mitochondrial genes analyzed (CYCS, ATP5B, IDH3b, NDUFB9), either under vehicle or rosiglitazone treatment. Interestingly, specific reduction of PGC-1β expression (70–80%) by the use of PGC-1β siRNA reduced the expression of mitochondrial genes in adipocytes by 20–40% under basal conditions, and also diminished the response to rosiglitazone (Figure 7B). These results strongly suggest that PGC-1β, but not PGC-1α, is the main regulator of mitochondrial gene expression in white adipocytes, and also that PGC-1β is the principal mediator of rosiglitazone-induced mitochondrial biogenesis in white adipocytes. Moreover, lack of PGC-1β significantly decreased basal and maximal adipocyte respiration, while lack of PGC-1α had minor or none effects (Figure 8). This supports our hypothesis that PGC-1β is crucial for mitochondrial function in white adipocytes.

## Discussion

The capacity of TZDs to ameliorate insulin resistance is typically attributed to their ability to activate PPARγ and promote adipogenesis [24], [25]. By enhancing the formation of new adipocytes, TZDs are thought to drive triacylglycerol storage in adipose tissues, reduce circulating lipid levels and prevent lipid accumulation and toxicity in other insulin-sensitive tissues [1]. However, a large body of evidence suggests that TZDs may also exert beneficial effects by promoting oxidative metabolism in adipose tissue [12], [17], [18], [26], a metabolic shift that has been hypothesized to be mediated by an increase in PGC-1α and subsequent PGC-1α action on mitochondrial gene expression and biogenesis. Consistent with this hypothesis, expression of exogenous PGC-1α in murine or human adipocytes promotes mitochondrial biogenesis and oxidative metabolism [27], [28]. Our results, however, argue against a role of endogenous PGC-1α as a mediator of both basal and rosiglitazone-induced mitochondrial biogenesis in WAT. Notably, even though rosiglitazone increased the expression of PGC-1α in Wt mice, the rosiglitazone-driven enhancement of mitochondrial gene expression, mitochondrial mass and mitochondrial enzymatic activities was similar in Wt and PGC-1α-FAT-KO mice. Consistent with the *in vivo* findings, suppression of PGC-1α by siRNA did not affect basal or rosiglitazone-induced expression of mitochondrial genes in 3T3-L1 adipocytes, nor did significantly affect their respiratory capacity.

The possibility that residual expression of PGC-1α in WAT of PGC-1α-FAT-KO mice due to the incomplete efficiency of genomic recombination could still drive mitochondrial gene expression cannot totally be ruled out. However, we think this is unlikely. First, the major bulk of the remaining PGC-1α expression in WAT of PGC-1α-FAT-KO mice could be attributed to other PGC-1α-expressing cell populations present in the tissue, such as endothelial cells [29]. Second, a comparable reduction of PGC-1α expression in muscle-specific PGC-1α knockout mice is sufficient to reduce the expression of mitochondrial genes in skeletal muscle [30], arguing against the capacity of low levels of PGC-1α to regulate mitochondrial gene expression. Likewise, the absence of an effect of PGC-1α on mitochondrial biogenesis and general mitochondrial gene expression in WAT cannot be attributed to the lack of PGC-1α transcriptional activity in WAT. Indeed, our findings show that adipose PGC-1α is required for the rosiglitazone-induced expression of brown adipocyte markers, such as UCP1 or CIDEA. The appearance of brown adipocytes in WAT can be induced by multiple signals, including long-term exposure to cold, hyperleptinemia and treatment with TZDs or β-adrenergic agonists [2], [10], [31], [32], [33]. PPARγ agonists and adrenergic stimulation act synergistically in inducing brown adipocytes in WAT [34]. Our study has been conducted at thermoneutrality, suggesting that rosiglitazone is sufficient to promote the appearance of brown adipocyte markers in the absence of β-adrenergic stimulation. This is consistent with recent findings showing that treatment of cultured primary white adipocytes with rosiglitazone induces the appearance of cells that express UCP1 and other brown fat markers in the absence of norepinephrine treatment [35], [36]. Thus, our findings suggest that adipose PGC-1α is important for the induction of UCP1 and other brown fat-specific genes not only in response to adrenergic signals, as seen in BAT [37], [38], but also to TZDs, as seen here in WAT.

Our study also provides evidence for a role of PGC-1β, a coactivator closely related to PGC-1α [39], [40], in basal and rosiglitazone-induced mitochondrial gene expression in white adipocytes. Our observations on the ability of PGC-1β to regulate basal mitochondrial gene expression and mitochondrial function in cultured 3T3-L1 white adipocytes are consistent with findings by others that attribute a role to PGC-1β in the control of mitochondrial gene expression in several cellular or animals models. For example, expression of exogenous PGC-1β in mouse skeletal muscle or in cultured muscle cells increases mitochondrial volume density and gene expression [41], [42], [43]. Conversely, mice with targeted deletion of PGC-1β display reduced expression of genes involved in mitochondrial energy metabolism in tissues such as brain, liver, heart, BAT or muscle [44], [45], [46]. In many of these tissues, PGC-1α and PGC-1β seem to have complementary, as well as redundant roles on mitochondrial gene expression [37], [44], [45], [46], [47], [48]. For instance, knockdown of either PGC-1α or PGC-1β in cultured brown adipocytes mildly affects basal expression of mitochondrial genes, while simultaneous lack of both PGC-1 proteins dramatically reduces expression of mitochondrial genes and severely impairs mitochondrial biogenesis and respiration [38]. In addition to PGC-1α and PGC-1β, the closely related coactivator PRC (PGC-1-Related Coactivator) has also been shown to regulate mitochondrial biogenesis, particularly in response to serum or proliferative signals [49], [50]. We did not observed an increase in the expression of PRC that could suggest a compensatory response to maintain mitochondrial mass in WAT of PGC-1α-FAT-KO mice. However, the fact that downregulation of PGC-1β by specific siRNA did not totally abolish expression of mitochondrial genes in 3T3-L1 adipocytes suggests that additional compensatory mechanisms may still exist in these cells. The potential involvement of PRC in mitochondrial biogenesis and the effect that lack of the three members of the PGC-1 family of coactivators has on adipocytes certainly deserve further exploration. Nevertheless, our findings suggest that WAT may represent a particular case in which PGC-1β carries a dominant role in the regulation of general mitochondrial gene expression, while PGC-1α acts specifically in the regulation of brown fat-specific genes in response to TZDs and, possibly, to other specific signals like cold exposure.

Interestingly, our results also suggest that PGC-1β mediates the induction of mitochondrial gene expression in response to rosiglitazone. In contrast to PGC-1α, PGC-1β expression is not induced by classic signals that promote mitochondrial biogenesis, such as physical exercise or exposure to low temperature [43]. Consequently, PGC-1β has been generally considered as a regulator of basal mitochondrial gene expression, whereas PGC-1α is thought to regulate gene expression in adaptation to signals of energy demand. However, our experiments show that PGC-1β is both induced by rosiglitazone and required for the rosiglitazone-induced increase of mitochondrial gene expression. Our results are in line with those recently published by Den et al. showing that TZDs require of PGC-1β to fully induce mitochondrial gene expression in 3T3-L1 adipocytes [51]. Together, these results suggest that PGC-1β can also participate in the dynamic regulation of mitochondrial gene expression in response to specific signals that promote oxidative metabolism.

The presence of brown adipocytes in WAT is thought to increase the capacity for energy expenditure and thus influence whole body energy balance [52]. Consistent with this notion, mouse strains with high content of brown adipocytes in WAT are highly resistant to the development of diet-induced obesity [53] and ectopic overexpression of UCP1 in WAT protects mice against obesity [54], [55]. Conversely, mice lacking UCP1 and raised at thermoneutrality show defects in diet-induced thermogenesis and become obese, particularly when on HFD [56]. The PGC-1α-FAT-KO mice did not show increased weight gain or adiposity at thermoneutrality. Notably, in the absence of rosiglitazone, UCP1 expression was similar in the PGC-1α-FAT-KO mice and Wt littermates, and consequently no differences in obesity would be necessarily expected. In the presence of rosiglitazone, PGC-1α-FAT-KO expressed lower levels of UCP1 compared to Wt mice; however, rosiglitazone treatment was for only 15 days. This length of treatment was sufficient to reveal an improvement in insulin sensitivity (in Wt and PGC-1α-FAT-KO mice) but may not have been long enough to reveal differences in thermogenesis and induce changes in body weight. Moreover, we did not observed any evidence for differential thermogenesis in BAT that could suggest any contribution of BAT to the phenotype observed in PGC-1α-FAT-KO mice. Indeed, first, expression of thermogenic genes (i.e. UCP1) was similar in BAT of Wt and PGC-1α-FAT-KO mice, especially under basal (non-treated) conditions. Second, the morphology of brown adipocytes in interescapular BAT, that at thermoneutrality and after a HFD feeding have adopted an appearance that make them practically indistinguishable of white adipocytes, was indicative of an inactive thermogenesis. Therefore, the contribution of BAT function to the reduced body weight of PGC-1α-FAT-KO seemed to be minimal.

Finally, it seems likely that PGC-1α in WAT has other, as of yet uncharacterized, functions that determine whole body energy balance. Consistent with this possibility, PGC-1α-FAT-KO mice showed an unexpected decreased body weight and adiposity, particularly when fed a HFD diet, and irrespectively of treatment with rosiglitazone. The mild reduction in fat mass seems to be the result of decreased triglyceride accumulation in adipocytes of PGC-1α-FAT-KO mice, and not due to impairments in adipocyte differentiation or changes in the expression of major genes involved in lipolysis, triglyceride esterification or lipid transport. Although PGC-1α function has been typically linked to regulation of catabolic pathways, a recent study has shown that overexpression of PGC-1α in skeletal muscle of mice activates *de novo* lipid synthesis [57]. In this line, overexpression of PGC-1α in C2C12 cells increases the expression of genes involved in lipid synthesis, such as acetyl-CoA carboxilase, fatty acid synthase or diacylglycerol acetyltransferase [58]. A similar, but milder, effect has been observed in liver when PGC-1α is overexpressed by adenoviral infection [59]. Interestingly, PGC-1α null mice also show lower body weight and decreased percent of body fat, especially when fed a HFD [36]. Moreover, low body weight due to decreased fat mass and reduced adipocyte size has also been observed in mice lacking ERRα [60], a nuclear receptor that mediates several of the PGC-1α actions on mitochondrial gene expression (reviewed in [61]). These results support the idea that PGC-1α may indeed affect lipid metabolism in more global ways than by simply enhancing fatty acid oxidation, although the metabolic pathways affected remain unknown.

In conclusion, our study shows that PGC-1α in WAT is not required to sustain basal or rosiglitazone-induced mitochondrial biogenesis. Moreover, the lack of adipose PGC-1α neither induces insulin resistance in mice nor it precludes the insulin-sensitizing effects of rosiglitazone. We also demonstrate that PGC-1α is important for the expression of UCP1 and other brown adipocyte-specific markers in WAT, revealing a role in the recruitment of brown adipocytes in WAT in response to rosiglitazone. Finally, our results suggest that PGC-1β regulates mitochondrial gene expression and function in adipocytes and mediates the effect of rosiglitazone on mitochondrial biogenesis. Future studies will need to address the *in vivo* function of adipose PGC-1β in mitochondrial biogenesis and adipocyte physiology.

## Materials and Methods

### Ethics statement

All procedures involving animals were performed in accordance with the institutional animal use and care guidelines of the Vall d'Hebron-Research Institute and approved by the Animal Experimentation and Ethics Committee of the Vall d'Hebron-Research Institute (ID 5/07 CEEA).

### Animals

To generate mice with floxed Ppargc1a alleles, a targeting vector was constructed by subcloning a *Bste II* - *SnaB I* (7,491 bp, containing exons 3, 4 and 5) and a *SnaB I* – *Xho I* (3,709 bp of intron 5) DNA fragment of a BAC genomic DNA clone encoding the murine *Ppargc1a* gene locus (Incyte Genomics, Palo Alto, CA) upstream and downstream, respectively, of a PGK-neomycin cassette flanked by two FRT sites and one LoxP site (gift of U. Mueller, TSRI, La Jolla, CA). An additional LoxP site was introduced by the cloning of an oligonucleotide at an *Apa I* site upstream of exon 4. The linearized targeting vector (Figure 1A) was electroporated into E14TG2a embryonic stems cells (derived from 129P2/OlaHsd mice) and two G418-resistant clones with the correct targeting event were selected and injected into C57BL/6 blastocysts. Germline-transmitting chimeras were mated with FLP deleter mice [62] to remove the PGK-neomycin selection cassette, generating mice with floxed exons 4 and 5 of the *Ppargc1a* locus. Mice with *Ppargc1a* floxed alleles were crossed 10 times to C57BL/6J mice, maintained in the C57BL/6J genetic background, and crossed to aP2-Cre [B6.Cg-Tg(Fabp4-cre)1rev/J] mice (Jackson Laboratory, Bar Harbor, ME) to generate adipose-specific deletion of exons 4 and 5. The deletion introduces a translation stop codon after exon 3. Mice with adipose-specific ablation of *Ppargc1a* gene are referred to as PGC-1α-FAT-KO mice.

Mice were raised and housed at thermoneutrality (30°C), so as to minimize the effects of impaired adaptive thermogenesis (due to PGC-1α ablation in brown adipocytes) on whole body energy homeostasis and enable the study of the role of PGC-1α specifically in white adipocytes.

When indicated, mice were fed a high fat diet (45% kcal fat, 35% kcal carbohydrate, 20% kcal protein) (Harlan Laboratories) for a period of 11 weeks, starting at 6 weeks of age. At 17 weeks of age, wild type (Wt) and PGC-1α-FAT-KO mice were treated with 10 mg/Kg rosiglitazone maleate (GlaxoSmithKline, Harlow, UK) or vehicle (ethanol) by oral gavage twice daily for 15 days. At the end of the treatment, mice were euthanized and tissues removed, weighed and stored for subsequent analysis.

### Serological analysis

Blood from PGC-1α-FAT-KO and Wt mice, treated with vehicle or rosiglitazone, was obtained after a 5-hour fasting from the saphenous vein and then centrifuged at 3,000 rpm during 5 min to obtain serum. Free fatty acids were determined with the NEFA-C kit (Wako Chemicals GmbH, Germany). Triglycerides and total cholesterol were measured by using commercial kits based on the Trinder method (FAR Diagnostics, Italy). For insulin determination, a mouse ELISA kit was used (ALPCO Diagnostics, NH, USA). Glucose levels were measured in blood using an ELITE glucometer (Bayer, Spain).

### Cell culture

3T3-L1 cells (ZenBio, USA) were cultured in DMEM media supplemented with 10% newborn calf serum (NCS) and differentiated to adipocytes as described previously [63]. For PGC-1α and PGC-1β knockdown, adipocytes were transfected on day 6 of differentiation with 50 nM of PPARGC1A or PPARGC1B ON-TARGETplus SMART pool siRNA using DharmaFECT 4 reagent (Thermo Fisher Scientific-Dharmacon), according to manufacturer's instructions. ON-TARGETplus Non-Targeting siRNA#2 was used as a negative control. After 24 h, media were replaced and cells were treated with 1 µM of rosiglitazone or an equivalent volume of DMSO and incubated for an additional 48 h. Cells were then harvested and RNA isolated for gene expression analysis.

### Gene expression

Total RNA was isolated from 3T3-L1 adipocytes or mouse tissues by using TRIzol reagent (Invitrogen, UK) according to the manufacturer's instructions. 400 ng of RNA were used to synthesize cDNA with SuperScript II reverse transcriptase (Invitrogen) and oligo(dT). Gene expression was assessed by real-time quantitative PCR using SYBR green and gene-specific primers in an ABI PRISM 7500 Sequence Detection System (Applied Biosystems, UK). Relative mRNA expression was calculated according to the 2^−ΔΔCT^ threshold cycle method, using cyclophilin as a reference gene, as described previously [64].

### Morphological analysis of adipose tissue

Subcutaneous WAT and interscapular BAT were fixed overnight in 4% formaldehyde. Then, tissue was dehydrated and embedded in paraffin for subsequent sectioning. Tissue sections (5–8 µm) were stained with hematoxylin/eosin. Image J software was used to measure adipocyte size from three animals of each experimental group. At least 300 cells from each animal were measured from 3 independent tissue sections.

### Mitochondrial DNA quantification

Total DNA was isolated from WAT by proteinase K digestion followed by phenol/chloroform extraction and ethanol precipitation. After isolation, DNA was treated with RNase A to eliminate any interference with RNA. Relative amounts of mitochondrial DNA (mtDNA) and nuclear DNA (nDNA) were determined by real-time quantitative PCR as previously described [64], using 2 ng of DNA as a template and specific primers for COXII (mtDNA) and RIP140 (nDNA).

### Citrate synthase activity

WAT was homogenized in extraction buffer (50 mM Tris/HCl, 1 mM MgCl2, 100 mM KCl, 250 mM sucrose and 30 mM 2-mercaptoethanol) and centrifuged at 1,000 g during 15 min at 4°C. The pellet containing cell debris and the upper lipid layer were carefully discarded and protein content in the supernatant was measured with a BCA Protein Assay Kit (Thermo Scientific-Pierce). Citrate synthase activity was then measured by the method of Srere [65]. Briefly, 10 µl of protein extract was added to 950 µl of reaction buffer (100 mM Tris/HCl pH = 8, 0.1% Trixton X-100, 0.11 mM DTNB, 0.25 mM Acetyl-CoA) and non-specific Acetyl-CoA hydrolase was measured by recording absorbance at 412 nm during 2 min at 25°C. Citrate synthase reaction was then initiated by adding oxalacetate to a final concentration of 0.28 mM and changes in absorbance at 412 nm were measured for 10 min. Citrate synthase activity was calculated after subtracting non-specific Acetyl-CoA hydrolase activity and was expressed in µmol/mg.min.

### Respiratory chain complexes activity

To measure the activity of the mitochondrial respiratory chain complexes, mitochondria-enriched fractions were first obtained from WAT as described by Pallotti and Lenaz [66]. After three cycles of freezing and thawing to disrupt mitochondrial integrity, protein content was measured as described above. Forty to sixty µg of protein were used to measure each complex activity by spectrophotometric methods as described by Degli [67] with minor modifications:

#### Complex I (NADH-ubiquinone oxidoreductase)

Reaction mixture was prepared by adding 50 µl of protein extract and 10 µl of 10 mM NADH to 950 µl of reaction buffer (50 mM KCl, 10 mM Tris-HCl, 1 mM EDTA, 2 mM KCN, 300 nM antimycin, pH = 7.4). The mixture was allowed to equilibrate for 1 min and then 5 µl of 10 mM decylubiquinone were added to start the reaction. The oxidation rate of NADH was measured on a spectrophotometer at 340 nm during 2 min at 30°C. Then, rotenone was added to the reaction and the rotenone-insensitive rate was measured for 10 min.

#### Complex II (succinate dehydrogenase)

Reaction was carried out at 30°C in 950 µl of potassium phosphate buffer (50 mM K-phosphate, 0.1 mM EDTA, 0.01% TritonX, 2 mM KCN, 300 nM antimycin, 10 µM rotenone) to which 50 µl of mitochondrial extract and 20 mM of succinate were added. After 5 min, reaction was started by the addition of 40 µM dichloroindophenol (DCIP) and 20 µM decylubiquinone and monitored at 600 nm during 3 min. Succinate dehydrogenase rate was calculated after subtracting non-specific enzymatic activity which was calculated by adding 0.5 mM of thenoyltrifluoroacetone (TTFA).

#### Complex II + III

Coupled succinate dehydrogenase and ubiquinone-cytochrome c reductase activities were measured as the rate of cytochrome c reduction using succinate as the electron donor. For this, 60 µl of mitochondrial extract were first incubated in reaction buffer (50 mM K-phosphate, 0.1 mM EDTA, 2 mM KCN, 10 µM rotenone, 20 mM succinate, pH = 7.4) during 5 min. The reaction was then initiated by the addition of 50 µM of oxidized cytochrome c and monitored at 550 nm during 3 min. Non-enzymatic rates were calculated after adding 0.2 µg of myxothyazol, an inhibitor of complex III.

#### Complex IV (cytochrome c oxidase)

Mitochondrial complex IV activity was determined by using a cytochrome c oxidase assay kit (Sigma, St. Louis, USA), according to manufacturer's instructions.

### Adipocyte respiration

Mature 3T3-L1 adipocytes were transfected with siRNA targeting PGC-1α or PGC-1β as described above. Forty-eight hours after transfection, cells were trypsinized, resuspended in DMEM media without serum and counted. Oxygen consumption was measured with a Clark-type oxygen electrode (Hansatech Instruments Ltd, Norfolk, UK) by using 2.5×10^5^ cells. Basal respiration was measured over a period of 5 min, after which 100 µM of the uncoupler CCCP was added in order to measure maximal respiration. Background was measured as the oxygen consumption rate after the inhibition of mitochondrial respiration with 1 mM of KCN.

### Western blot

For UCP1 detection, 40 µg of WAT protein were resolved by SDS/PAGE, transferred to a PVDF membrane and probed with an antibody against UCP1 (ABCAM) or α-tubulin (Cell Signaling) as previously described [64]. 10 µg of BAT protein from Wt mice housed at 21°C were used as positive control.

### Statistical analysis

Results are expressed as mean ± SEM. Unpaired Student's *t* test was used to assess the significance of differences between experimental groups.

## Supporting Information

Figure S1 **Morphology of BAT at thermoneutrality.** Brown adipocyte morphology of Wt and PGC-1α-FAT-KO mice raised at thermoneutrality and fed a standard diet was analyzed in histological sections of BAT stained with haematoxilin/eosin.
(TIF)

Figure S2 **Morphology of BAT of mice fed a high fat diet and treated with rosiglitazone.** Brown adipocyte morphology of Wt and PGC-1α-FAT-KO mice raised at thermoneutrality and fed a high fat diet for 11 weeks was analyzed in histological sections stained with haematoxilin/eosin after treatment with vehicle or rosiglitazone.
(TIF)

Figure S3 **Whole body glucose tolerance and insulin sensitivity.** (A) Glucose tolerance test (GTT) was performed on 12-h fasted mice. Blood glucose levels were measured at 0, 20, 30, 60, 90 and 120 min after an intraperitoneal injection of glucose (1 g/Kg). (B) Insulin tolerance test (ITT), performed after a 5 h-fast. Glucose levels in blood were measured at 0, 20, 30, 60, 90 and 120 min after an intraperitoneal injection of insulin (0.75 U/Kg) (n = 6–9 animals/group).
(TIF)
