# Supplementary figures and images for: Rosiglitazone-Induced Mitochondrial Biogenesis in White Adipose Tissue Is Independent of Peroxisome Proliferator-Activated Receptor γ Coactivator-1α
Source: PLoS One. 2011 Nov 7;6(11):e26989. doi: 10.1371/journal.pone.0026989 (PMC3210129; doi:10.1371/journal.pone.0026989)

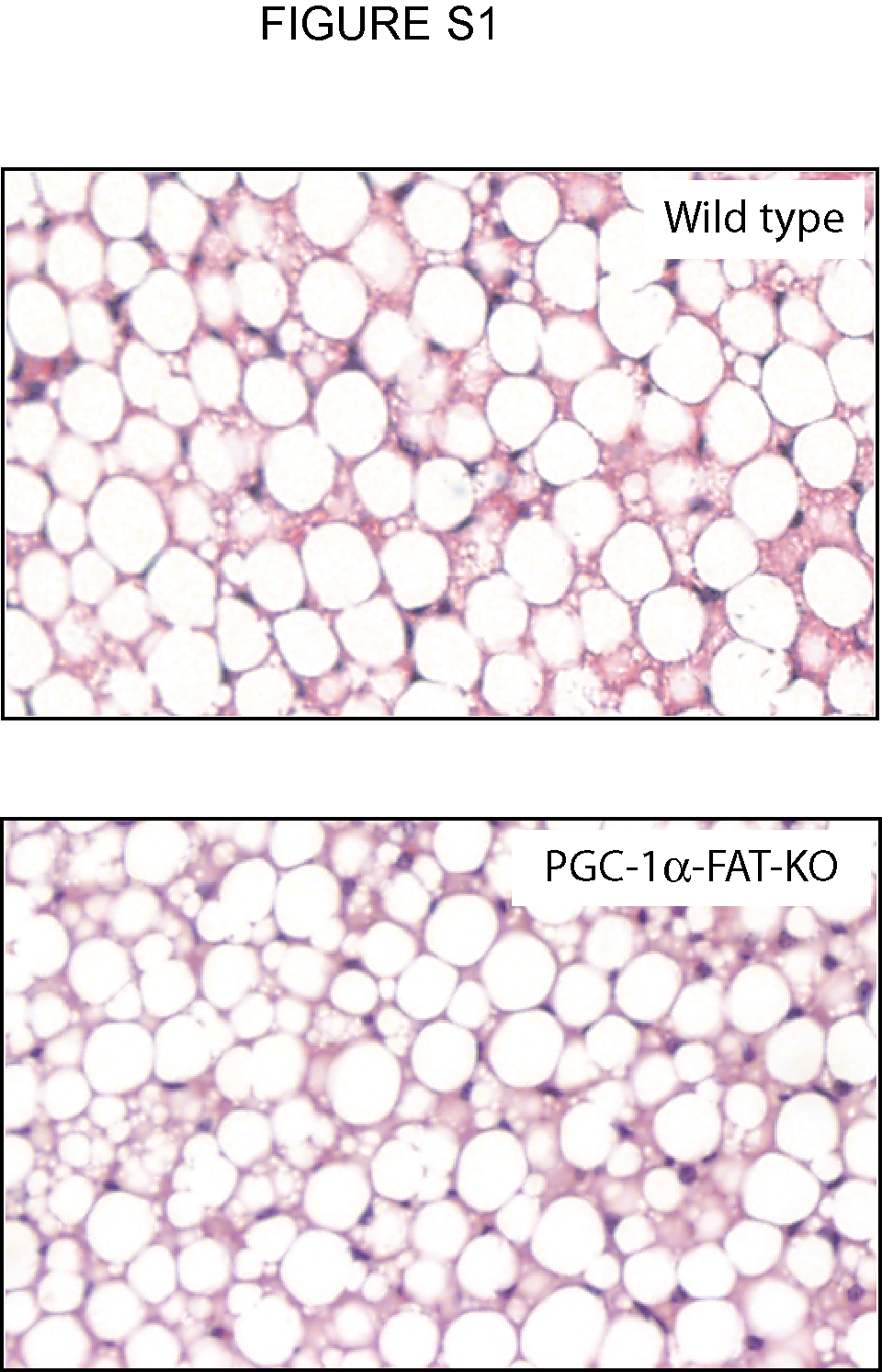

Supplement: Figure S1 — Morphology of BAT at thermoneutrality. Brown adipocyte morphology of Wt and PGC-1α-FAT-KO mice raised at thermoneutrality and fed a standard diet was analyzed in histological sections of BAT stained with haematoxilin/eosin. (TIF) [file pone.0026989.s001.tif]

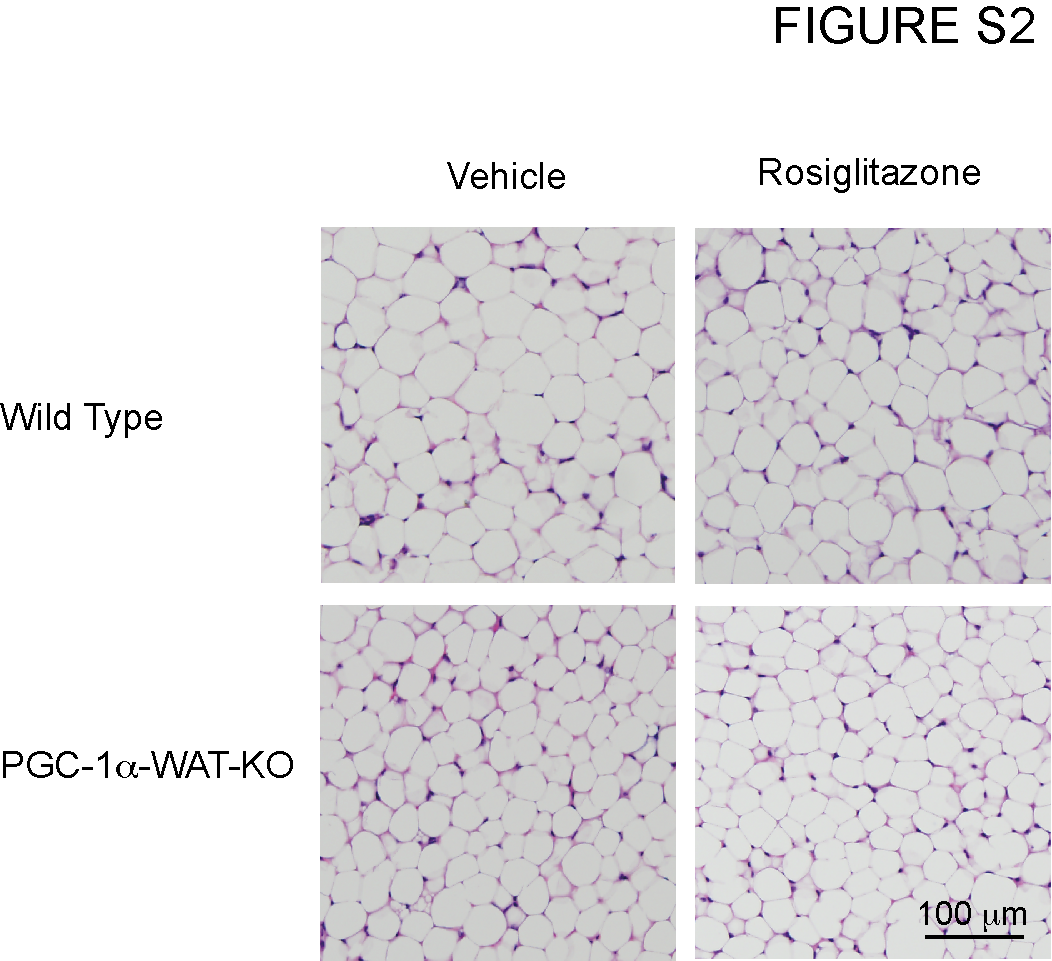

Supplement: Figure S2 — Morphology of BAT of mice fed a high fat diet and treated with rosiglitazone. Brown adipocyte morphology of Wt and PGC-1α-FAT-KO mice raised at thermoneutrality and fed a high fat diet for 11 weeks was analyzed in histological sections stained with haematoxilin/eosin after treatment with vehicle or rosiglitazone. (TIF) [file pone.0026989.s002.tif]

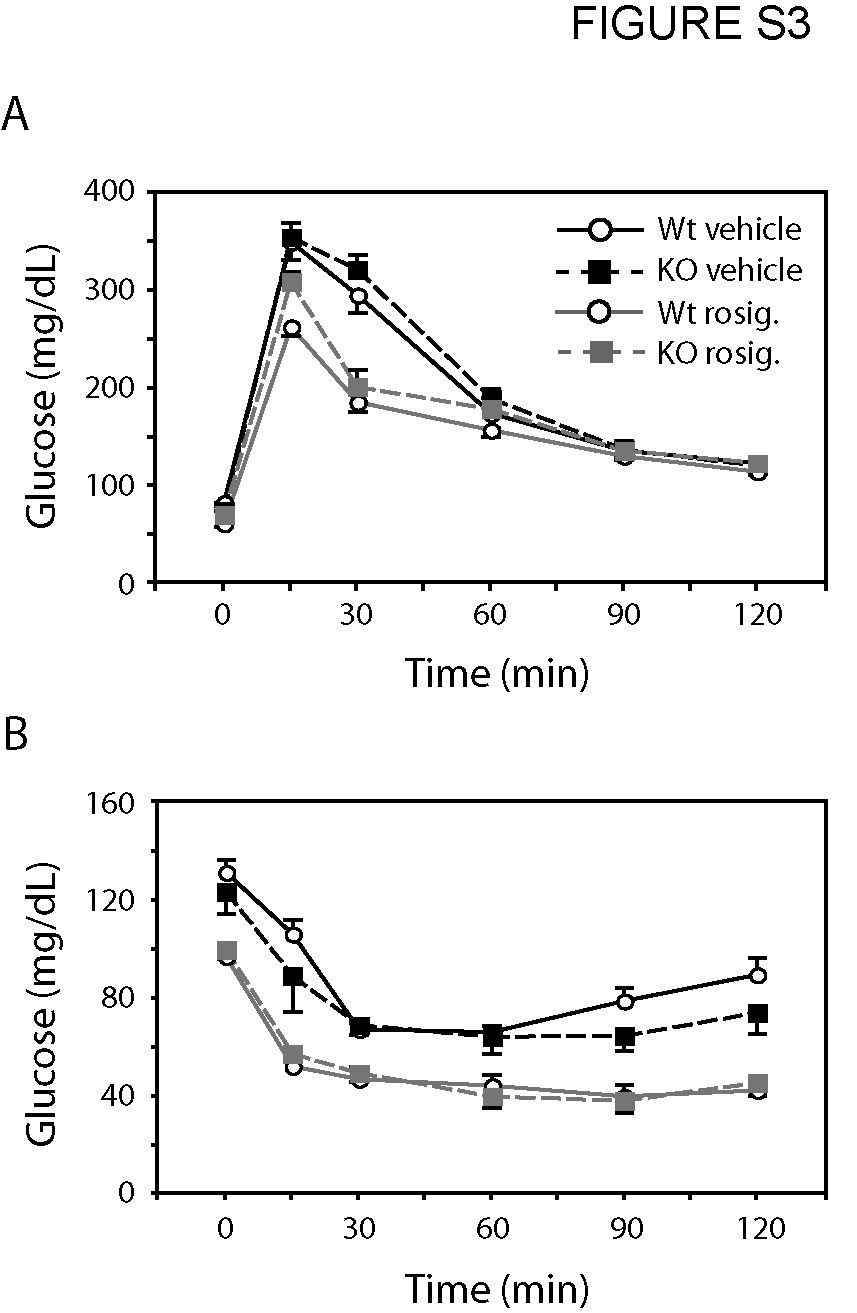

Supplement: Figure S3 — Whole body glucose tolerance and insulin sensitivity. (A) Glucose tolerance test (GTT) was performed on 12-h fasted mice. Blood glucose levels were measured at 0, 20, 30, 60, 90 and 120 min after an intraperitoneal injection of glucose (1 g/Kg). (B) Insulin tolerance test (ITT), performed after a 5 h-fast. Glucose levels in blood were measured at 0, 20, 30, 60, 90 and 120 min after an intraperitoneal injection of insulin (0.75 U/Kg) (n = 6–9 animals/group). (TIF) [file pone.0026989.s003.tif]
